# Reducing the Excess Activin Signaling Rescues Muscle Degeneration in Myotonic Dystrophy Type 2 *Drosophila* Model

**DOI:** 10.3390/jpm12030385

**Published:** 2022-03-02

**Authors:** Jing Deng, Xin-Xin Guan, Ying-Bao Zhu, Hai-Tao Deng, Guang-Xu Li, Yi-Chen Guo, Peng Jin, Ran-Hui Duan, Wen Huang

**Affiliations:** 1 Center for Medical Genetics, School of Life Sciences, Central South University, Changsha 410017, China; dengjing@sklmg.edu.cn (J.D.); guanxinxin@sklmg.edu.cn (X.-X.G.); zhuyingbao@sklmg.edu.cn (Y.-B.Z.); denghaitao@sklmg.edu.cn (H.-T.D.); liguangxu@sklmg.edu.cn (G.-X.L.); guoyichen@sklmg.edu.cn (Y.-C.G.); duanranhui@sklmg.edu.cn (R.-H.D.); 2 Hunan Key Laboratory of Medical Genetics, Central South University, Changsha 410017, China; 3 Hunan Key Laboratory of Animal Models for Human Diseases, Central South University, Changsha 410017, China; 4 Department of Medical Genetics, Yueyang Maternal and Child Health-Care Hospital, Yueyang 414000, China; 5 Department of Human Genetics, School of Medicine, Emory University, Atlanta, GA 30322, USA; peng.jin@emory.edu

**Keywords:** chemical screen, *Drosophila*, myotonic dystrophy type 2, TGF-β pathway

## Abstract

Expanded non-coding RNA repeats of CCUG are the underlying genetic causes for myotonic dystrophy type 2 (DM2). There is an urgent need for effective medications and potential drug targets that may alleviate the progression of the disease. In this study, 3140 small-molecule drugs from FDA-approved libraries were screened through lethality and locomotion phenotypes using a DM2 *Drosophila* model expressing 720 CCTG repeats in the muscle. We identified ten effective drugs that improved survival and locomotor activity of DM2 flies, including four that share the same predicted targets in the TGF-β pathway. The pathway comprises two major branches, the Activin and BMP pathways, which play critical and complex roles in skeletal development, maintenance of homeostasis, and regeneration. The *Drosophila* model recapitulates pathological features of muscle degeneration in DM2, displaying shortened lifespan, a decline in climbing ability, and progressive muscle degeneration. Increased levels of p-smad3 in response to activin signaling were observed in DM2 flies. Decreased levels of activin signaling using additional specific inhibitors or genetic method ameliorated climbing defects, crushed thoraxes, structure, and organization of muscle fibers. Our results demonstrate that a decrease in activin signaling is sufficient to rescue muscle degeneration and is, therefore, a potential therapeutic target for DM2.

## 1. Introduction

Myotonic dystrophy (DM) is one of the most common dominantly inherited neuromuscular disorders in adults. It affects at least 1 in 8000 people worldwide [1]. There are two genetically distinct types, myotonic dystrophy type 1 (DM1) and myotonic dystrophy type 2 (DM2), both of which are caused by different unstable noncoding repeat expansions. DM1 is caused by a trinucleotide CTG-repeat expansion in the 3′UTR of the *DMPK* gene [2,3,4]. DM2 is caused by a tetranucleotide CCTG-repeat in the first intron of the *CNBP* (also known as *ZNF9*) gene [5]. Previous studies have shown that DM1 and DM2 are toxic RNA-mediated spliceopathies. In DM patients, transcription of expanded repeats of toxic RNA sequesters MBNL protein family from their normal functions and alters pre-mRNA splicing in a range of downstream effector genes, resulting in pleiotropic phenotypes [6,7]. DM affects various types of muscles, particularly the skeletal muscles characterized by myotonia, muscle weakness, and wasting [8]. Although the same pathogenic mechanisms have led to similar clinical symptoms in DM1 and DM2, they present a number of distinguishable features. In DM1, trinucleotide repeat lengths in affected patients’ range between 50 and 4000 CTG repeats, and the severity correlates with the expansion size [9]. In contrast to DM1, the age of onset and disease severity is not linked to the length of the repeat expansion in DM2. The expansions can be up to 44 kb (11,000 CCTG) in affected DM2 individuals [10]. There are great heterogeneity and large phenotypic variability among affected DM2 individuals. These may be partly explained by differences in their repeated sequences. Besides the sharing of the MBNL family of proteins, there are increasing activities of CUG-binding protein (CUGBP1/CELF1) and dsRNA-dependent protein kinase (PKR) in DM1 [10,11,12,13]; however, this was not found in DM2. In addition, RBFOX competes with MBNL for binding to CCTG repeats rather than CTG repeats, which releases some MBNL and alleviates the disease [14].

There is still no effective treatment for DM2. Most therapeutic studies are currently focused on DM1, and thus DM2 requires more attention. Many small-molecule compounds have been found to ameliorate DM phenotype in the DM1 models by targeting toxic RNA, MBNL levels, mis-splicing, and CEFL1 levels. Based on the biochemical characteristics of CUG repeats, some triaminotriazine-based and 2,9-diaminoalkyl-1,10-phenanthroline (DAP)-based small molecules were designed to compete with MBNL for CUG [15,16,17]. ISOX, vorinostat, and histone deacetylase could upregulate MBNL1 levels to rescue DM phenotypes [18]. Manumycin A was identified to improve splicing abnormalities [19]. CEFL1 level could be restored by Ro–31–8220 [20]. Pentamidine treatment was shown to be effective in DM1 but had no effect on the DM2 *Drosophila* model [21,22]. There are differences between repetitive DNA sequence and RNA-binding proteins in DM1 and DM2. Most of these compounds that are effective in DM1 have not been verified in the DM2 model, thus the effectiveness of these compounds in DM2 needs further study. 

High-throughput screening has been developed in *Drosophila* models such as Fragile X syndrome, Alexander disease and Friedreich’s ataxia to screen for potential therapeutic targets [23,24,25]. Several drugs have been successfully identified to mitigate the neurotoxicity of disease models. Nancy M. Bonini created a *Drosophila* model that expresses pure, uninterrupted CCUG-repeat expansions ranging from 16 to 720 repeats in length, which may recapitulate key features of human DM2 including RNA repeat-induced toxicity, ribonuclear foci formation, and changes in alternative splicing [26]. To identify potential therapeutic agents for DM2, we tested an FDA-approved small molecule drug library in this DM2 *Drosophila* model. Four compounds that inhibited transforming growth factor (TGF-β) signaling were screened and suppressed the phenotype of DM2 flies. The TGF-β pathway mediates diverse biological processes, which is an evolutionarily conserved signal transduction module in *Drosophila*. The pathway is generally divided into two branches defined by the utilization of receptor Smads (R-Smads): the activin branch and the bone morphogenic protein (BMP) branch [27]. It plays important roles in different physiological processes in the skeletal muscle, such as growth, differentiation, homeostasis, impaired regeneration, and atrophy, highly induced in a variety of inherited and acquired neuromuscular disorders [28]. 

In this study, we conducted large-scale drug screening using the *Drosophila* model for DM2 with 3140 small-molecule drugs from FDA-approved libraries. From this screening, four compounds that inhibited transforming growth factor (TGF-β) signaling were identified as the most active compounds which rescued muscle degeneration. The DM2 *Drosophila* model that expresses 720 CCTG repeats only in adult muscles could simulate key aspects of DM2 human with an obvious decline in the climbing ability, reduced lifespan, and progressive muscle degeneration. p-smad3, in response to activin signaling, is elevated in the DM2 flies. Genetic suppression and specific inhibitors of activin signaling could indeed ameliorate the declined locomotor activity and progressive muscle degeneration.

## 2. Materials and Methods

### 2.1. Drosophila Stocks

The smox RNAi strains used in most of this study (Stock ID #26756, genotype y^1^ v^1^; P{TRiP.JF02320}attP2/TM3, Sb1) were obtained from Bloomington *Drosophila* Stock Center (BDSC, Indiana University, Bloomington, IN, USA). This smox RNAi is targeted against nucleotides 1506–1933 of the smox coding sequence. A second smox RNAi strain (Stock ID #41670, genotype y^1^ sc* v1 sev^21^; P{TRiP.HMS02203}attP40) was obtained from BDSC, targeted against nucleotides 898–9189 of the smox coding sequence. The med RNAi strains used in most of this study were Stock ID #31928 (genotype: y^1^ v^1^; P{TRiP.JF02218}attP2), obtained from BDSC. This med RNAi is targeted against nucleotides 2612–3017 of med coding sequence. The second med RNAi strain (Stock ID #43961, genotype: y^1^ sc* v^1^ sev^21^; P{TRiP.GL01313}attP40), from BDSC, targeted against nucleotides 1845–1865 of med coding sequence. The results utilizing the second smox and med RNAi strains are shown in the Supplementary Materials. Two muscle-specific drivers used are 24B-GAL4 (a gift from Prof. Zhuohua Zhang) and Mef2-GAL4 (BDSC, Stock ID #27390). UAS-(CCTG) _n_ flies were a gift from Prof. Nancy M. Bonini (Boston University School of Medicine, Boston, MA, USA). We used the Mef2-Gal4; tub-Gal80ts to study the role of TGF-β signaling in the adult muscle of the DM2 *Drosophila* model. The binding affinity of Gal4 to UAS sequences can be selectively controlled by the temperature-sensitive Gal80 repressor [29]. The target flies after emergence were placed at 29 °C in order to activate the expression of UAS-RNAi and UAS-(CCTG)_n_. Other fly stocks were maintained at 25 °C in a standard medium.

### 2.2. Drug Treatment

About 3140 compounds approved by the US Food and Drug Administration (FDA) were individually tested in triplicate. The compounds were placed in 96-well polypropylene (PP) microtiter plates. The plates were stored at −20 °C. Stock solutions of the tested compounds (10 mM in DMSO), or similar volumes of DMSO for control conditions, were mixed with food medium to a final concentration of 40 µM. SB431542 (Med Chem Express, Monmouth Junction, NJ, USA) and SIS3 (Med Chem Express) were dissolved in DMSO (Sigma-Aldrich, Saint Louis, MI, USA) and added to standard food at 60 µM and 20 µM, respectively. DMSO alone was used as control.

### 2.3. Climbing Assays

To assess the climbing velocity, groups of ten male flies aged to a certain number of days were transferred into climbing-ability test plastic tubes and incubated for 1 h at room temperature to allow for environmental adaptation and anesthesia awareness. Tubes were tapped until all flies were at the bottom and the time that it took for the fifth fruit fly to climb 15 cm was recorded. For each genotype, approximately 30 flies were tested. Each experiment was repeated three times.

### 2.4. Lifespan Analysis

A minimum of 100 newly enclosed flies were used for lifespan analysis. Males were separated and 10 flies were placed per vial. The flies were transferred to fresh vials every alternate day and the number of dead flies was recorded. The survival distribution of the two genotypic groups was compared using the log-rank (Mantel–Cox) test.

### 2.5. Immunolabeling and Confocal Microscopy

To observe the individual indirect flight muscles (IFMs), heads and abdomens were removed and the remaining body was fixed in 4% paraformaldehyde (PFA) for 1 h at room temperature (RT) or overnight at 4 °C. Then, the muscular tissue was obtained from the dissected thorax in 0.3% PBST and blocked for 1 h in 0.3% PBST with 10% normal goat serum for 1 h at RT. Primary antibodies were rabbit anti-cleaved caspase 3 (1:200, Cell Signaling Technology, Danvers, MA, USA), rabbit anti-p-mad (a gift from Prof. Peter ten Dijke) and anti-p-smad3 (1:200, Abcam, Cambridge, UK), secondary antibodies conjugated to DyLight 488 or 649 (Jackson Immuno Research, West Grove, PA, USA) were used at 1:200. The muscle fibers were labeled with rhodamine-phalloidin (1:1000, Thermo Fisher Scientific, Bedford, MA, USA) and nuclei were counterstained with DAPI (1 µg/mL; Sigma Aldrich, Saint Louis, MI, USA). The samples were observed with Zeiss LSM880 laser scanning confocal microscopy, with a Plan-Apochromat 63x/1.4 Oil DIC M27 objective. 

### 2.6. Drosophila Paraffin Section

The histological sections of the muscle were prepared from wax-embedded material. Flies were fixed in Carnoy’s solution (ethanol, trichloromethane, and glacial acetic acid in proportion 6:3:1, respectively) at 4 °C overnight. The sample was dehydrated using increasing concentrations of ethanol for 5 min (40%, 40%, 70%, 100%, and 100%). Next, the flies were incubated in MB, MB + paraffin solution (V:V = 1:1), paraffin I and paraffin II for 30 min in each at 62 °C. The collar was rapidly relocated into a plastic box and filled with melted (65 °C) paraffin and placed at room temperature overnight. The sections were stained with H&E (hematoxylin and eosin) and analyzed using light microscopy (DM5000 B; Leica Microsystems, Buffalo Grove, IL, USA) [30]. 

### 2.7. Western Blot

In order to analyze the phosphorylation levels of R-Smad, thoraxes from adult *Drosophila* were used to extract protein. The antibodies used for protein detection on Western blots were anti-phosphorylated-mad (a gift form Prof. Peter ten Dijke), anti-phosphorylated-smad3 (1:1000, Abcam, Cambridge, UK) and anti-α-tubulin (1:5000, Sigma-Aldrich, Saint Louis MI, USA). All Western blots were repeated at least three times with similar results. The intensity of each band was quantified using an image analysis software.

### 2.8. Statistical Analysis

All results are presented as mean ± SEM. Statistical analysis was performed with one-way ANOVA for multiple comparisons or two-way ANOVA, as indicated in the individual figure legends. *p*-values ≤ 0.05 were considered as statistically significant as indicated by asterisks: * *p* ≤ 0.05, ** *p* ≤ 0.01 and *** *p* ≤ 0.001.

## 3. Results

### 3.1. Drugs Targeting the TGF-β Pathway Could Rescue Lethality and Locomotion of DM2 Fly Model

In order to obtain small molecule drugs that could alleviate symptoms of DM2 fly model, we screened 3140 FDA-approved small drug molecules. The lethality and locomotion phenotypes were utilized to identify chemical modifiers of the CCUG-induced toxicity through two-rounds of screening. Expressing 720 CCTG repeats in the muscle under 24B-GAL4 driver is lethal in the pupa stage. We crossed the muscle-specific mef2-GAL4 driver to UAS-CCTG_720_ and counted the survival rate of offspring upon each drug treatment. The primary screen identified 20 positive drugs which can improve the survival rate of the offspring. Muscle, as part of the multisystem involvement, is the most severely affected tissue in DM2 patients. Expressing 720 CCTG repeats under Mef2-GAL4 driver, another muscle-specific driver, shows weaker toxicity and their offspring can survive to adulthood. The crawling ability can be reliably used for second screening and 10 positive drugs were identified. Among the 10 drugs, 4 effective drugs were discovered through two-round screening and they have common predicted targets in the TGF-β pathway (Table 1).

**Figure 1:**
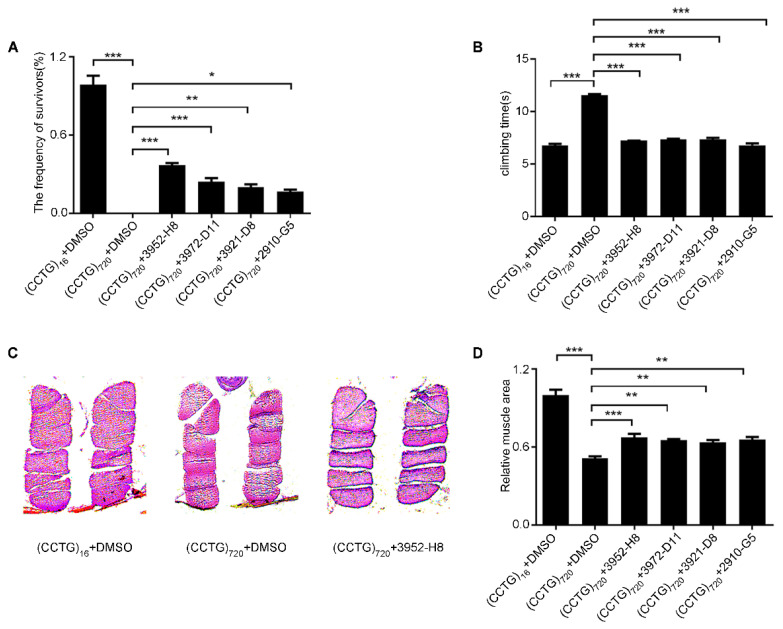
The studied drugs rescued lethality and motor function in the DM2 fly model. (**A**) The four compounds targeting the TGF-β pathway rescued the lethality of 24B-GAL4 > (CCTG)_720_. (**B**) Upon drug treatment separately, climbing ability of Mef2-GAL4 > (CCTG)_720_ measured at day 5. (**C**) IFM transversal sections of Mef2-GAL4 > (CCTG)_720_ with H&E (hematoxylin and eosin) staining at day 5. Images were taken at 100× magnification with a Leica DM5000. (**D**) Quantitative analysis of muscle areas of Mef2-GAL4 > (CCTG)_720_ with the administration of the drug. All statistical models were tested using one-way ANOVA, * *p* < 0.05; ** *p* < 0.01; *** *p* < 0.001.

DM2 flies were improved in terms of survival and locomotion after treatment of the four candidate drugs including 2910-G5, 3921-D8, 3952-H8, and 3972-D11. The flies expressing 720 CCTG repeats in muscle under 24B-GAL4 driver can survive to adulthood and the survival rate increases to around 40% (Figure 1A). Upon drug treatment, the climbing ability of DM2 flies under the Mef2-GAL4 driver was corrected to around 7 s compared to DMSO-treated with 13 s (Figure 1B). Further, smaller tears and larger muscle areas were seen in the paraffin sections in DM2 flies (Figure 1C,D). There was no significant effect of DMSO treatment. Four drugs targeting the TGF-β pathway may effectively mitigate the muscle toxicity of DM2.

### 3.2. Flies Expressing CCTG Repeats in the Muscle at the Adult Stage Showed Shorter Lifespan and Muscle Degeneration

**Figure 2:**
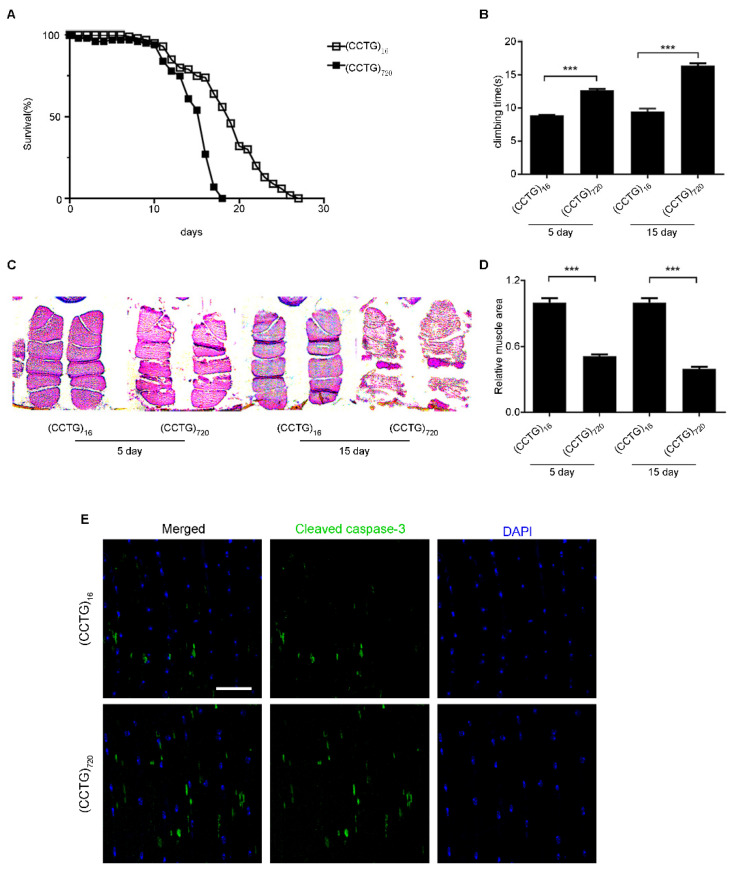
Flies expressing CCTG repeats in the muscle at adult stage show shorter lifespan, declined climbing ability, and muscle degeneration. (**A**) Flies expressing 720 CCTG repeats in adult muscle have a significantly shorter lifespan in comparison to those expressing 16 CCTG repeats. (**B**) Climbing ability of flies measured at days 5 and 15. (**C**) IFM transversal sections of flies with H&E (hematoxylin and eosin) staining at days 5 and 15. The flies of (CCTG)_720_ displayed muscle degeneration. Images were taken at 100× magnification with a Leica DM5000. (**D**) Quantitative analyses of muscle areas. (**E**) Confocal images of the myofiber of IFM in 15-day-old male flies showed increased cleaved caspase 3 in the muscle of (CCTG)_720_. Cleaved caspase 3 (green), DAPI (blue). Bar equals 50 μm. All statistical models were tested using two-way ANOVA, *** *p* < 0.001.

The TGF-β pathway plays an important role in the early development of skeletal muscle. Nevertheless, DM2 is an adult-onset disease without congenital form, unlike DM1, and most of the clinical features of DM2 patients appear in adulthood. A DM2 fly model using the Mef2-GAL4/tub-Gal80ts system expressed the expanded CCTG repeats in adult muscle, avoiding developmental effects and better simulating the characteristics of DM2. The 720 CCTG repeats expression was activated as soon as the flies shifted to 29 °C, while the expression of CCTG repeats was suppressed at 19 °C. The crossed flies were raised at 19 °C until male offspring were collected and moved to a restrictive temperature of 29 °C. The lifespan of (CCTG)_720_ was significantly shortened compared to (CCTG)_16_ and the mean lifespan was only 15 days (Figure 2A). The flies of (CCTG)_720_ displayed a weakness in climbing ability at five days and more severe crawling disability could be observed when aged to 15 days (Figure 2B). We also discovered obvious muscle vacuoles and tears of DM2 flies in paraffin slice at five days. The muscle of these flies aged to 15 days was phenotypically severe, demonstrating irregular shape, visibly bigger holes, and tears, even fragmentation of the entire muscle mass (Figure 2C). The flies expressing 16 CCTG repeats showed normal climbing ability and intact muscle structure. The (CCTG)_720_ muscle area was lost by at least 50% compared to (CCTG)_16_, though muscle area statistical (Figure 2D). Cleaved caspase 3 was marked to examine apoptosis in adult muscle by immunoreactivity that detected an increase in the adult muscle of (CCTG)_720_ in comparison to (CCTG)_16_ (Figure 2E). There was no difference between (CCTG)_720_ and (CCTG)_16_ when the flies were kept at 19 °C (Supplementary Figure S1). The fly model generalized key features of human DM2 and demonstrated progressive muscle degeneration.

### 3.3. The Activin Signaling Is Increased in the DM2 Fly Model

**Figure 3:**
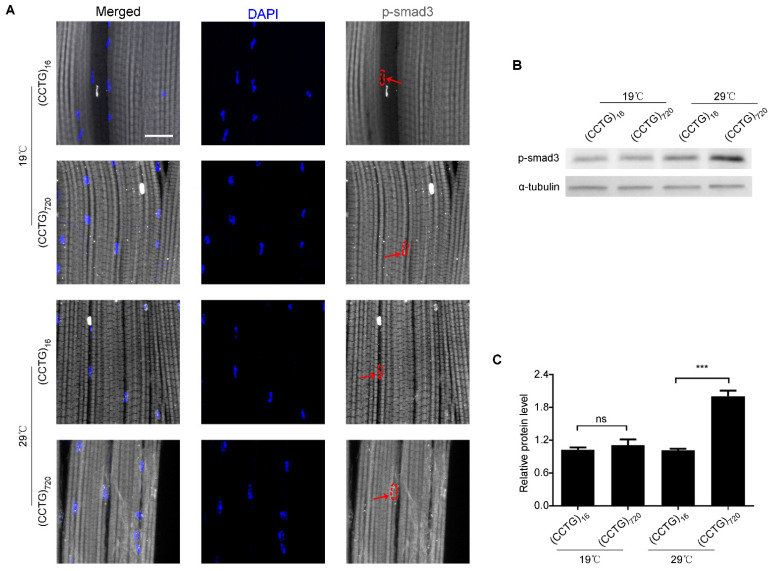
P-smd3 is increased when expressing CCTG repeats in the adult muscle of flies. (**A**) Confocal images of the myofiber of IFM in 15-day-old male flies at 19 °C (the expression of CCTG repeats was suppressed) or 29 °C (the expression of CCTG repeats was activated. P-smad3 in the nucleus marked with red arrows. Increased p-smad3 in adult muscle cell nucleus of (CCTG)_720_ was detected compared to (CCTG)_16_ at 29 °C. p-smad3 (gray), and DAPI (blue). Bar equals 20 μm. (**B**) Western blotting was used to measure p-smad3 levels in DM2 flies that were increased compared with the control flies at 29 °C. (**C**) Bar graphs show changes in the quantitative analysis of relative smad3 phosphorylation level after (CCTG)_720_ expression. Data from three independent experiments are shown as means ± SEM (*p*-values were determined by two-way ANOVA, *** *p* < 0.001).

The phenotypic changes within IFM of DM2 flies are alleviated by drugs targeting TGF-β signaling. In *Drosophila*, TGF-β signaling is classically divided into the BMP signaling and activin signaling. P-mad responds to BMP signaling. Another p-Smad3 responds to activin signaling. To explore the functional role of the TGF-β signaling pathway in DM2 degenerated muscle, we examined TGF-β signaling by probing the phosphorylation of its mad and smad3. Transgenic male flies expressing the (CCTG)_720_ or (CCTG)_16_ repeat expansion under the Mef2-Gal4/tub-Gal80ts system driver were collected and aged for 15 days at 29 °C. Increased p-smad3 in the adult muscle cell nucleus of (CCTG)_720_ was detected comparing to (CCTG)_16_ (Figure 3A). Western blot analyses were used to detect the levels of p-mad and p-Smad3 (Figure 3B). A significant increase in the p-Smad3 levels was observed in (CCTG)_720_ flies compared to (CCTG)_16_ flies (Figure 3B), while p-mad was not changed significantly (Supplementary Figure S2). There is no difference in p-Smad3 levels between (CCTG)_720_ and (CCTG)_16_ flies aged for 15 days when the expression of CCTG repeats was suppressed at 19 °C. Our results showed that activin signaling was increased obviously in DM2 flies.

### 3.4. Reducing Activin Signaling Could Rescue Muscle Degeneration

**Figure 4:**
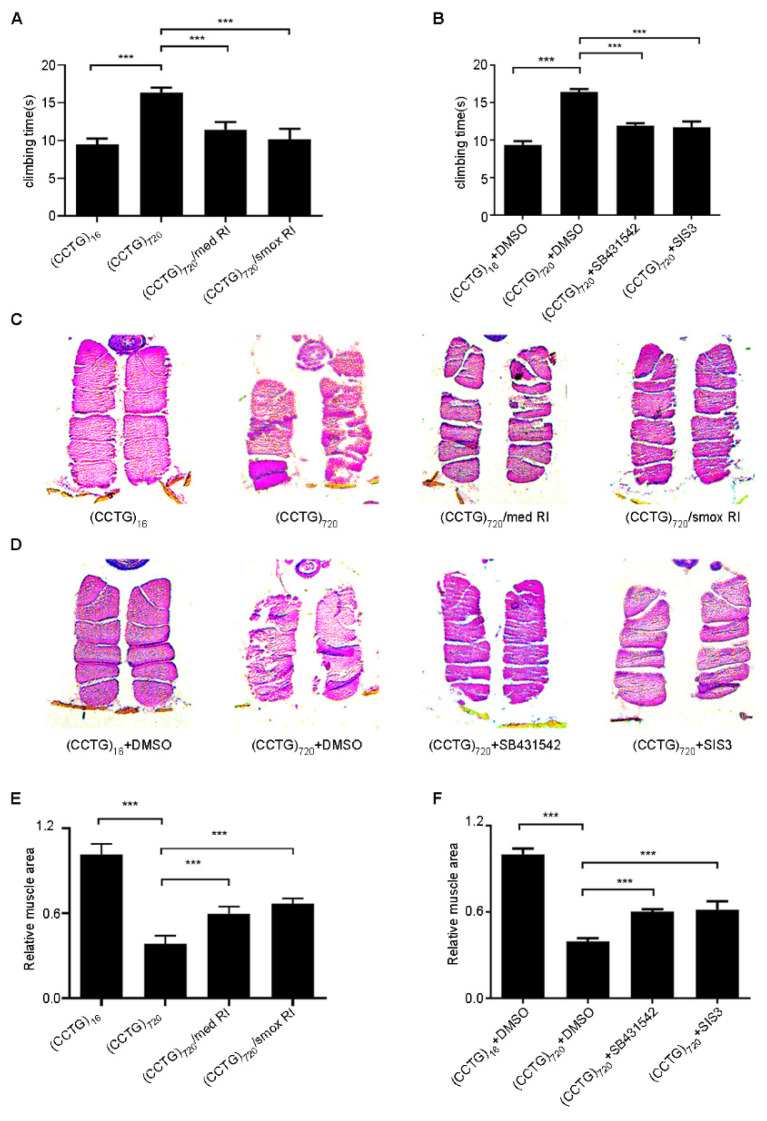
Reducing the activin signaling could improve climbing ability and crushed thoraxes of DM2 flies. (**A**,**B**) Climbing ability of flies measured at day 15. Attenuating key molecules, *med* or *smox*, from the activin signaling ameliorated climbing defects in (CCTG_)720_ flies (**A**). (CCTG)_720_ flies treated with specific inhibitors of activin signaling, the concentrations of SB431542 and SIS3: 60 µM, 20 µM (**B**). (**C**,**D**) IFM transversal sections of flies with H&E (hematoxylin and eosin) staining at day 15. Images were taken at 100× magnification with a Leica DM5000. Reducing *med* or *smox* could suppress crushed thoraxes in (CCTG)_720_ flies (**C**). (CCTG)_720_ flies treated with SB431542 or SIS3. The concentrations of SB431542 and SIS3: 60 µM, 20 µM (**D**). (**E**) Quantitative analyses of muscle areas of (CCTG)_720_ flies crossed with *med* or *smox* RNAi lines (15 days after eclosion). (**F**) Quantitative analyses of muscle areas of (CCTG)_720_ flies exposed to SB431542 or SIS3 (15 days after eclosion). The concentrations of SB431542 and SIS3 were 60 µM and 20 µM, respectively. All statistical models were tested using one-way ANOVA, *** *p* < 0.001.

Increased p-Smad3 has been observed in DM2 flies. To determine whether TGF-β signaling was one of the effective targets for DM2, the genetic suppression was introduced to DM2 flies. In *Drosophila*, *med* is the common-mediator of Smad (co-Smad), which is required for transducing signals of all TGF-β superfamily members. We used the Mef2-GAL4/tub-Gal80ts system to express the expanded CCTG repeat and to decrease the *med* level in the adult muscle at 29 °C. The climbing ability of DM2 flies with reductive *med* were significantly improved from a simultaneously 15-day-aged cohort of DM2 flies (Figure 4A). Muscle disorganization in the magnified thorax sections of DM2 flies by paraffin slices was largely inhibited by reducing *med*, and the muscle area was increased by 20% (Figure 4C,E). Suppression of *smox* in DM2 adult muscle, a *Drosophila* homologous gene to smads 2 and 3 for the activin signaling, resulted in a great improvement in the walking and muscle defect (Figure 4A,C). The loss of muscle area in (CCTG)_720_ was increased to 60–70 percent of (CCTG)_16_ (Figure 3E). The same results were verified with another *med* and *smox* RNAi line (Supplementary Figure S3). The knockdown efficiency of two *med* RNAi strains and two *smox* RNAi strains were 40–60%, (data not shown).

Specific inhibitors targeting activin signaling were also used to verify if muscle relevant changes in DM2 flies are due to activin signaling. SB431542 is a potent and selective TGF-β inhibitor and SIS3 is a specific inhibitor of smad3 [31,32,33]. We treated DM2 flies under the Mef2-Gal4/tub-Gal80ts system driver with SIS3 or SB431542 for 15 days at 29 °C. The concentrations were previously determined to be appropriate. We observed that inhibition of activin signaling with SIS3 or SB431542 rescue partly crushed thoraxes and climbing defects in the DM2 flies (Figure 4B,D,F). Inhibitors were verified with Western blot (Supplementary Figure S4). Two lines of evidence verified that reducing the activin signaling could ameliorate progressive muscle degeneration of DM2 flies.

### 3.5. Reducing Activin Signaling Could Rescue Myofiber Defects

**Figure 5:**
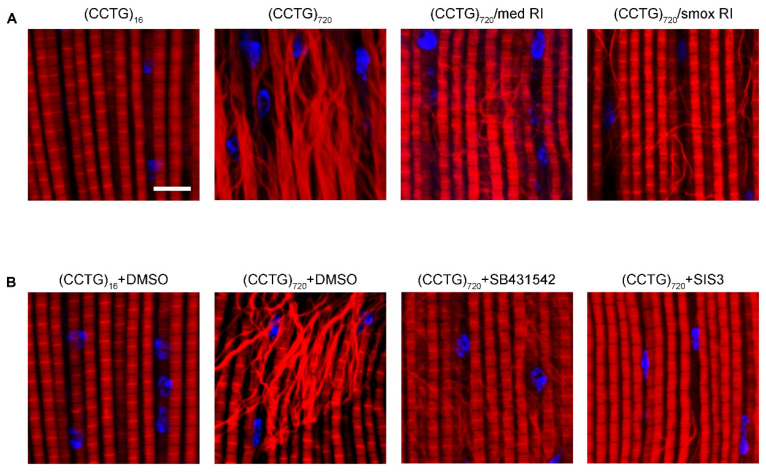
Reducing the activin signaling could rescue myofiber defects of DM2 flies. (**A**,**B**) Confocal images of the myofiber of 15-day-old male flies. DAPI (blue) and phalloidin (red). Bar equals 10 μm. Attenuating med or smox ameliorated myofiber defects in (CCTG)_720_ flies (**A**). (CCTG)_720_ flies exposed to SB431542 and SIS3. The concentrations of SB431542 and SIS3 were 60 µM and 20 µM, respectively. Attenuating med or smox ameliorated myofiber defects in (CCTG)_720_ flies (**B**).

Voids and tears can be observed in the adult muscle of DM2 by paraffin incisions. In order to evaluate the degree of muscle involvement, we observed indirect flight muscle (IFM) fibers of these flies at high resolution labeled by phalloidin. Muscle destruction was apparent at day 15 of age at 29 °C. The muscle fibers of DM2 flies were slim and disorganized. The swelling and deformation of muscle fibers were frequently observed (Figure 5A). The muscle fibers were unwounded in the (CCTG)_16_ flies in the same condition.

In order to investigate whether decreased activin signaling acts against defects of muscle fibers in DM2 flies, the suppression of *med* and *smox* in DM2 flies under the Mef2-GAL4/tub-Gal80ts system driver was promoted by genetic suppression and drug treatment. The shape and structure of muscle fibers were improved through genetic suppression at 15 days (Figure 5A). Using inhibitors targeting the activin signaling could also ameliorate myofiber morphology defects (Figure 5B). The decline of the activin signaling could improve the mass of muscles, structure, and organization of muscle fibers in the DM2 *Drosophila* model.

## 4. Discussion

There is no effective treatment available for myotonic dystrophy type 2. In our study, we screened 3140 FDA-approved drugs using a *Drosophila* model expressing 720 CCTG repeats specifically in the muscle. Ten compounds were identified as capable of inhibiting muscle toxicity in DM2 flies after two rounds of screening. Among the ten compounds, four drugs share the same predicted target-TGF-β pathway, a decrease in which was found to efficiently mitigate lethality and improve locomotion in the DM2 *Drosophila* model.

TGF-β signaling plays an important role in skeletal muscle development and homeostasis. Among the multiple systems involved, the groups of muscles are the most affected in DM2 [34]. To overcome the effects of the TGF-β pathway in the developmental stage of flies, the expanded CCTG repeats were conditionally expressed in the adult muscle. The *Drosophila* model displays a shortened lifespan and declined locomotor activity. More importantly, muscle degeneration recapitulates the key aspects of DM2 phenotypes. Splitting muscle fibers, reduced fiber size, swelling and deformation of muscle fibers may reflect the severity of myofiber involvement. Apoptosis occurred more frequently in aged adults. The conditional model is adapted for studying the role of TGF-β signaling in DM2 *Drosophila*. 

The TGF-β signaling pathway is divided into two branches—BMP signaling and activin signaling. Many lines of evidence suggest that the TGF family is involved in the control of muscle repair [35]. Increased activin signaling is seen in response to repetitive injury in muscle disease. Acute injuries alone, in the absence of disease, also induced enhanced activin activity [36]. Experimental results showed that increased TGF-β activity could suppress satellite cell activation, affect myocyte differentiation and lead to tissue fibrosis in response to skeletal muscle injury, which could accelerate muscle degeneration [37,38,39]. Muscular dystrophy arises from ongoing muscle degeneration and insufficient regeneration [40]. The fly muscle with muscular dystrophy is mainly characterized by a degenerative process, which allowed us to examine the myogenic component of injury response. Enhanced p-smad3, in response to activin signaling, was discovered in the muscle of adult DM2 flies. Nuclear accumulation of p-Smad3 in myofiber nuclei is likely a consequence of repetitive and ongoing muscle damage. Diminishing activin signaling could suppress muscle degeneration in DM2 flies. These results imply that increased activity of the activin signaling may play an important role in the process of muscle degeneration in DM2 flies. 

Excess TGF-β signaling has been involved in the pathogenesis in animal models and human muscular dystrophy. Several researchers have reported that activation of Smad3 is associated with cardiac dysfunction, myocardial fibrosis, and cardiomyocyte apoptosis [41]. The strong activation of TGF-β1 has been discovered in cardiac specimens of DM1 patients [42]. In addition, dystrophic muscle biopsies showed that TGF-β1 was gathered at injured muscle fibers of DMD patients [43]. The mdx mice, the most universally used Duchenne muscular dystrophy (DMD) model, showed improved muscle function and decreased fibrosis when activin signaling was reduced [44,45,46]. Similar results were observed in a DMD *Drosophila* model, the muscular dystrophy model with the deletion in the gene encoding sarcoglycan (Sgcd) that is a key component for maintaining muscle membrane integrity along with dystroglycan and dystrophin. The myonuclei most proximal to the region of disruption displayed the greatest amount of TGF-β1 activity, partial reduction of the co-SMAD med, or the smox responding to activin signaling corrected both heart and muscle dysfunction in Sgcd mutants [47]. Recently, an antagonist of activin signaling-related ligands, follistatin (FS), was used in a Becker muscular dystrophy (BMD) proof-of-principle clinical trial with encouraging results. Histological changes showed reduced endomysial fibrosis, reduced central nucleation, and more normal fiber size distribution with muscle hypertrophy [48]. The above findings support that TGF-β signaling may be a potential therapeutic target in muscular dystrophy.

In summary, we conducted chemical screening and identified compounds targeting TGF-β signaling that could suppress muscular toxicity caused by CCTG repeats. In particular, we showed that reducing excess activin signaling could suppress muscle degeneration in DM2 flies (a scheme of activin signaling pathway in DM2 fly is shown in Supplementary Figure S5). Our study presents activin signaling as a potential therapeutic target for DM2, which requires further evaluation in other DM2 mammalian models.

## Figures and Tables

**Table 1:** Four compounds identified to rescue the lethality and locomotion of DM2 *Drosophila*.

No.	PubChem CID	Molecular Formula	Chemical Name
3952-H8	5382764	C_10_H_8_N_2_O_2_	2-[(E)-2-nitroethenyl]-1H-indole
3972-D11	239794	C_21_H_14_ClN_3_O_4_S	4-[(3-chloro-1,4-dioxonaphthalen-2-yl)amino]-N-pyridin-2-ylbenzenesulfonamide
3921-D8	364428	C_9_H_6_OS_2_	5-thiophen-2-ylthiophene-2-carbaldehyde
2910-G5	319846	C_37_H_47_NO_14_	23-(dimethylamino)-4,8,12,22,24-pentahydroxy-1,12-dimethyl-10-(3,4,5-trimethoxy-4,6-dimethyloxan-2-yl) oxy-20,25-dioxahexacyclo [19.3.1.0^2,19^.0^5,18^.0^7,16^.0^9,14^] pentacosa-2,4,7(16),8,14,18-hexaene-6,17-dione

## Data Availability

The authors affirm that all data necessary for confirming the conclusions of the article presented in the article are represented fully within the article. The data presented in this study are available in article or Supplementary Material.

## Supplementary Materials

The following supporting information can be downloaded at: https://www.mdpi.com/article/10.3390/jpm12030385/s1, Figure S1. Flies show normal climbing ability and intact muscle structure when the expression of 720 CCTG repeats was suppressed. (A) Climbing ability of flies measured at days 5 and 15. (B) Quantitative analyses of muscle areas. (C) IFM transversal sections of flies with H&E (hematoxylin and eosin) staining at days 5 and 15. Images were taken at 100× magnification with a Leica DM5000. (D) Confocal images of the myofiber of 15-day-old male flies. DAPI (blue) and phalloidin (red). Bar equals 10 μm. All statistical models were tested using two-way ANOVA, * *p* < 0.05; ** *p* < 0.01; *** *p* < 0.001; Figure S2. P-mad is not changed when expressing CCTG repeats in the adult muscle of flies. (A) Confocal images of the myofiber of IFM in 15-day-old male flies at 19 °C (the expression of CCTG repeats was suppressed) or 29 °C (the expression of CCTG repeats was activated. p-mad (green), and DAPI (blue). Bar equals 20 μm. (B) Western blotting was used to measure p-mad levels in DM2 flies that were not changed compared with the control flies at 29 °C. (C) Bar graphs show changes in the quantitative analysis of relative mad phosphorylation level after (CCTG)720 expression. Data from three independent experiments are shown as means ± SEM (*p*-values were determined by two-way ANOVA, ** *p* < 0.01 and *** *p* < 0.001); Figure S3. Reducing the activin signaling could improve climbing ability and crushed thoraxes of DM2 flies. (A) Climbing ability of flies measured at day 15. Attenuating key molecules, med or smox, from the activin signaling ameliorated climbing defects in (CCTG)720 flies. (B) Quantitative analyses of muscle areas of (CCTG)720 flies crossed with med or smox RNAi lines (15 days after eclosion). (C) IFM transversal sections of flies with H&E (hematoxylin and eosin) staining at day 15. Reducing med or smox could suppress crushed thoraxes in (CCTG)720 flies. Images were taken at 100× magnification with a Leica DM5000. (D) Confocal images of the myofiber of 15-day-old male flies. DAPI (blue) and phalloidin (red). Bar equals 10 μm. Attenuating med or smox ameliorated myofiber defects in (CCTG)720 flies. All statistical models were tested using one-way ANOVA, * *p* < 0.05; ** *p* < 0.01; *** *p* < 0.001; Figure S4. SB431542 and SIS3 could specifically reduce the phosphorylation levels of smad3. (A) Inhibitors were verified with Western blot. (B) Bar graphs show changes in the quantitative analysis of relative smad3 phosphorylation level after inhibitors treatment in 15 day. The phosphorylation of smad3 was downregulated by nearly 50% after treatment with SB431542 or SIS3. Data from three independent experiments are shown as means ± SEM (*p*-values were determined by two-way ANOVA, ** *p* < 0.01 and *** *p* < 0.001); Figure S5. Scheme of activin signaling in DM2 fly model. Reducing excess activin signaling by genetic suppression or pharmacological inhibition could suppress muscle degeneration in DM2 flies.
