# Supplementary figures and images for: Reducing the Excess Activin Signaling Rescues Muscle Degeneration in Myotonic Dystrophy Type 2 Drosophila Model
Source: J Pers Med. 2022 Mar 2;12(3):385. doi: 10.3390/jpm12030385 (PMC8948895; doi:10.3390/jpm12030385)

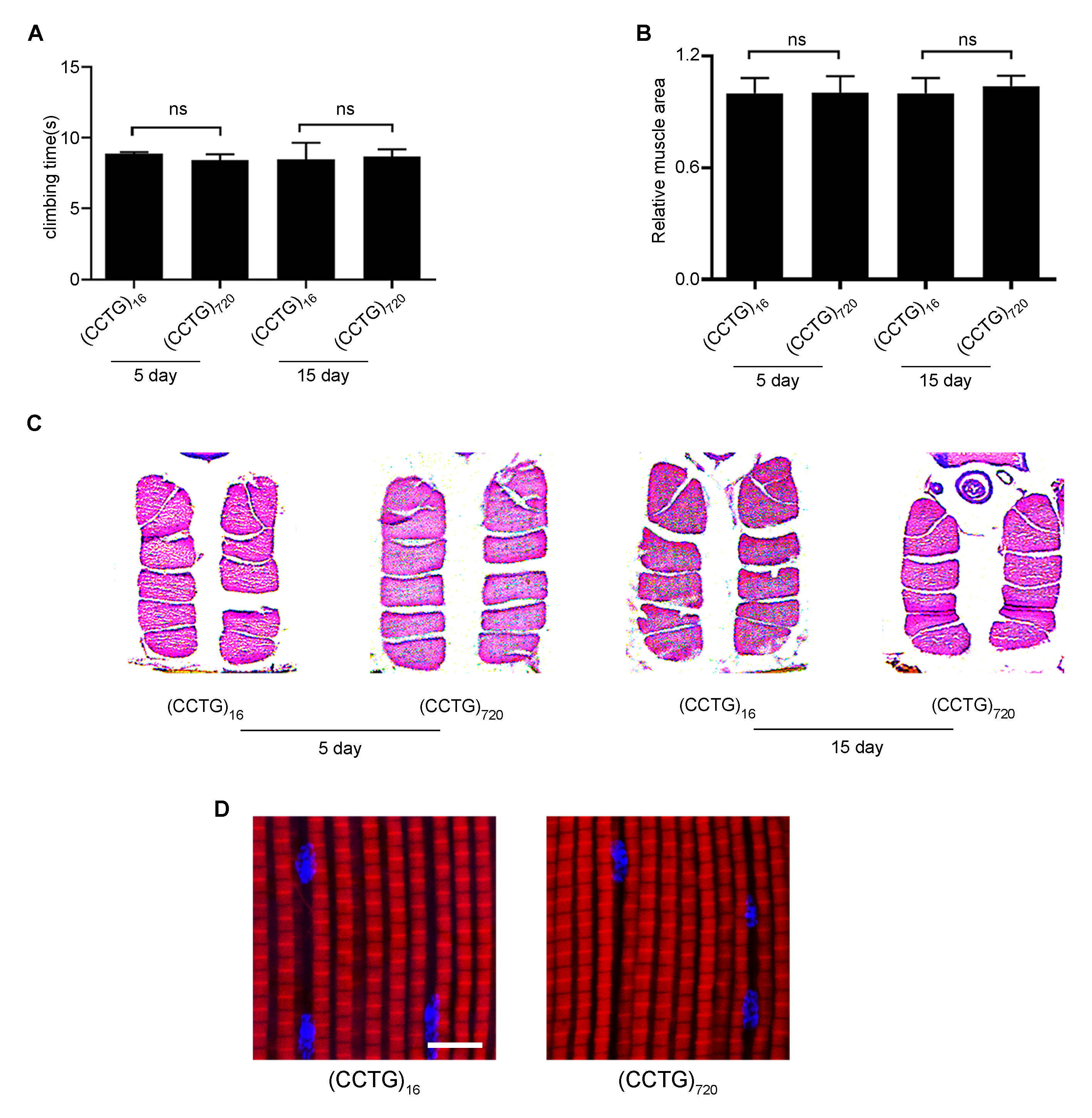

Supplement: Supplementary file 1 [file jpm-12-00385-s001.zip › Figure-S1.tif]

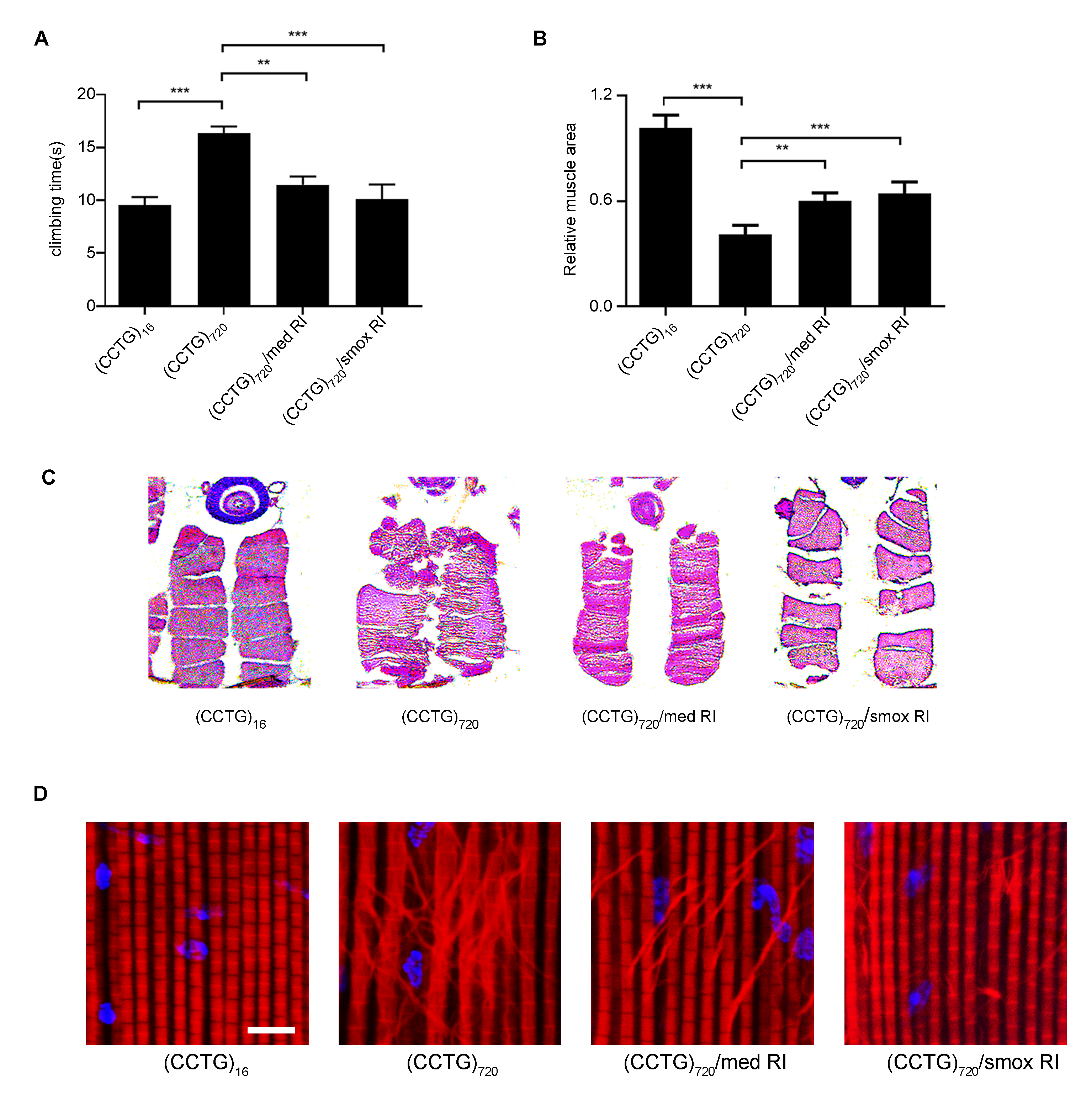

Supplement: Supplementary file 1 [file jpm-12-00385-s001.zip › Figure-S3.tif]

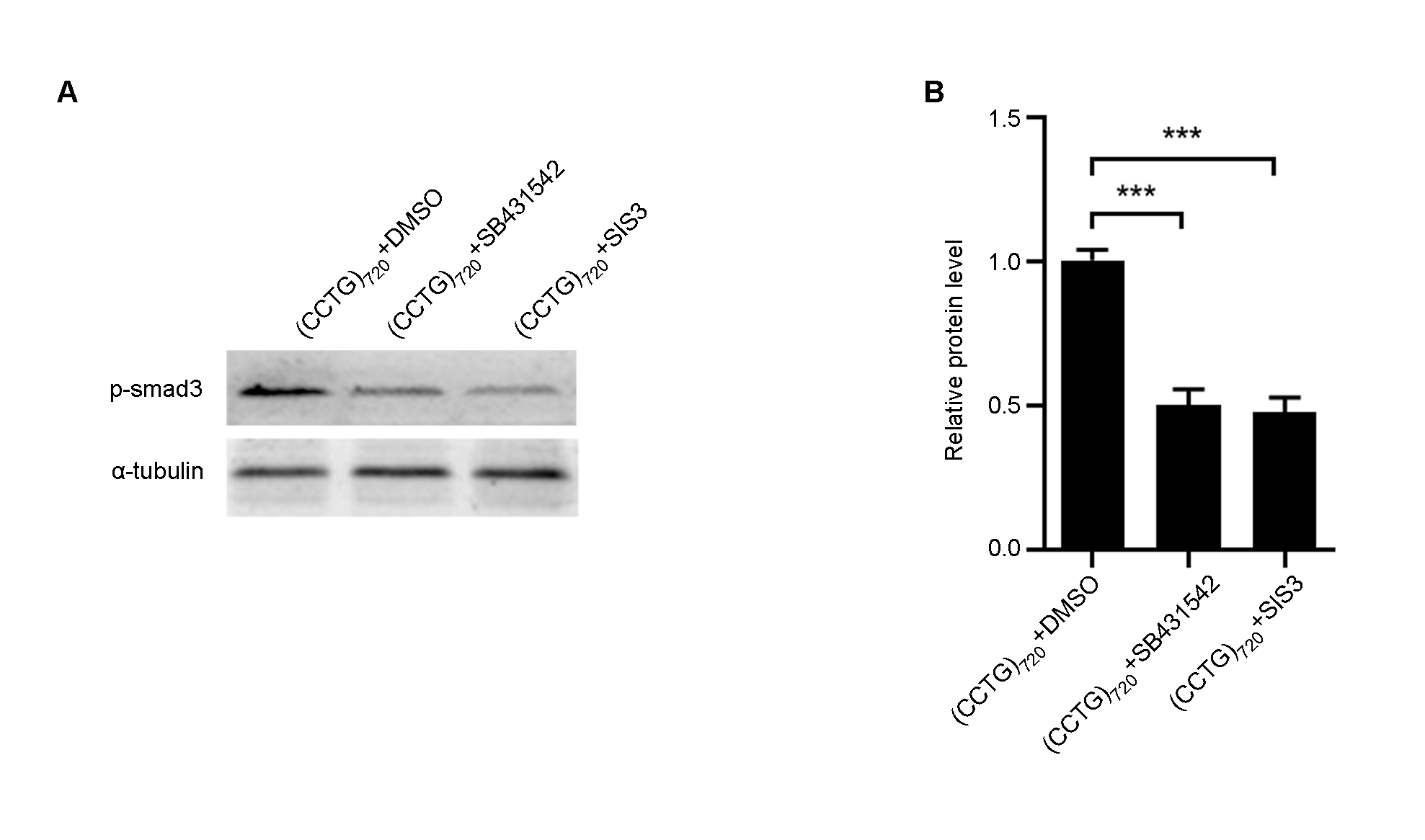

Supplement: Supplementary file 1 [file jpm-12-00385-s001.zip › Figure-S4.tif]

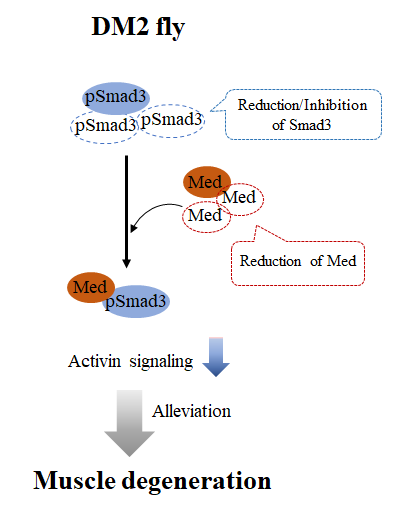

Supplement: Supplementary file 1 [file jpm-12-00385-s001.zip › Figure-S5.tif]
